# Implementation of the "FASTHUG" concept decreases the incidence of ventilator-associated pneumonia in a surgical intensive care unit

**DOI:** 10.1186/1754-9493-2-3

**Published:** 2008-02-12

**Authors:** Thomas J Papadimos, Sandra J Hensley, Joan M Duggan, Sadik A Khuder, Marilyn J Borst, John J Fath, Lauri R Oakes, Debra Buchman

**Affiliations:** 1 Department of Anesthesiology, University of Toledo College of Medicine, 3000 Arlington Avenue, Toledo, USA; 2 Department of Prevention and Infection Control, University of Toledo Medical Center, 3000 Arlington Avenue, Toledo, USA; 3 Department of Medicine, University of Toledo College of Medicine, 3000 Arlington Avenue, Toledo, USA; 4 Department of Surgery, University of Toledo College of Medicine, 3000 Arlington Avenue, Toledo, USA; 5 Department of Quality and Clinical Safety, University of Toledo Medical Center, 3000 Arlington Avenue, Toledo, USA; 6 Department of Nursing Research and Evaluation, University of Toledo College of Nursing, 3000 Arlington Avenue, Toledo, USA

## Abstract

**Background:**

Ventilator-associated pneumonia (VAP) is a leading cause of morbidity and mortality in critically ill patients. The Institute for Healthcare Improvement 100,000 Lives Campaign made VAP a target of prevention and performance improvement. Additionally, the Joint Commission on Accreditation of Health Organizations' 2007 Disease Specific National Patient Safety Goals included the reduction of healthcare-associated infections. We report implementation of a performance improvement project that dramatically reduced our VAP rate that had exceeded the 90^th ^percentile nationally.

**Methods:**

From 1 January 2004 to 31 December 2005 a performance improvement project was undertaken to decrease our critical care unit VAP rate. In year one (2004) procedural interventions were highlighted: aggressive oral care, early extubation, management of soiled or malfunctioning respiratory equipment, hand washing surveillance, and maximal sterile barrier precautions. In year two (2005) an evaluative concept called FASTHUG (daily evaluation of patients' feeding, analgesia, sedation, thromboembolic prophylaxis, elevation of the head of the bed, ulcer prophylaxis, and glucose control) was implemented. To determine the long-term effectiveness of such an intervention a historical control period (2003) and the procedural intervention period of 2004, i.e., the pre-FASTHUG period (months 1–24) were compared with an extended post-FASTHUG period (months 25–54).

**Results:**

The 2003 surgical intensive care VAP rate of 19.3/1000 ventilator-days served as a historical control. Procedural interventions in 2004 were not effective in reducing VAP, p = 0.62. However, implementation of FASTHUG in 2005, directed by a critical care team, resulted in a rate of 7.3/1000 ventilator-days, p ≤ .01. The median pneumonia rate was lower after implementation of FASTHUG when compared to the historical control year (p = .028) and the first year after the procedural interventions (p = .041) using follow-up pairwise comparisons. The pre-FASTHUG period (2003–2004, months 1–24) when compared with an extended post-FASTHUG period (2005–2007, 25–54 months) also demonstrated a significant decrease in the VAP rate, p = .0004. This reduction in the post-FASTHUG period occurred despite a rising Severity of Illness index in critically ill patients, p = .001.

**Conclusion:**

Implementation of the FASTHUG concept, in the daily evaluation of mechanically ventilated patients, significantly decreased our surgical intensive care unit VAP rate.

## Introduction

Ventilator-associated pneumonia (VAP) is a leading cause of morbidity and mortality in critically ill patients [1]. It is a form of hospital-associated pneumonia that occurs 48 hours or more after tracheal intubation and mechanical ventilation of a patient [2]. It occurs in 9%–27% of all intubated, mechanically ventilated patients and increases hospital stays by 7 to 9 days at an excess cost of up to $40,000 per patient [3-5]. The Institute for Healthcare Improvement (IHI) 100,000 Lives Campaign has made VAP a target of prevention and performance improvement in intensive care units [6]. In addition to the IHI's targeting of VAP, the Joint Commission on Accreditation of Healthcare Organizations' (JCAHO) 2007 Disease-Specific National Patient Safety Goals (goal 7) included the reduction of the risk of health care-associated infections [7]. Our surgical intensive care unit (SICU) VAP rate of 19.3/1000 ventilator-days was high, at the 90th percentile for SICUs according to the 2004 National Nosocomial Infection Surveillance (NNIS) system [8]. We implemented a performance improvement project over 2 years to reduce our SICU VAP rates. A successful decrease in the SICU VAP rate was realized in the second year of the project with the addition of the FAST-HUG concept (daily evaluation of feeding, analgesia, sedation, thromboembolic prevention, head of bed elevation, ulcer prophylaxis, and glucose control in critically ill patients) in the SICU [9].

## Methods

From 1 January 2004 to 31 December 2005 a performance improvement project was undertaken in the SICU to decrease the incidence of VAP. The occurrences of VAP were documented prospectively in the hospital's infection control database, but the review of the performance improvement project data was retrospective. Institutional Review Board approval was received for publication of the data. The SICU is a ten-bed unit in a 319-bed university medical center that averages 667 admissions per year. The SICU cares for trauma, general surgery, and all surgical sub-specialty patients. Project years 2004 and 2005 each had their SICU VAP rate compared to the historical control year, 2003. Sixty days before year 1 of the project (November 2003) an intensivist-led critical care team model was instituted in the SICU. The critical care team consisted of faculty physicians, anesthesiology and surgery residents, medical students, nurses, a pharmacist, and respiratory therapists. In year 1 (2004) of the project *procedural *interventions were highlighted. Aggressive oral care using chlorhexidine mouthwash, an early extubation strategy, changing respiratory equipment only when visibly soiled or malfunctioning, and aggressive enforcement of hand-washing and barrier protection methods for central line placement were introduced and applied to all mechanically ventilated patients.

Chorhexidine mouthwash, which had not been used consistently, was now used every 12 hours on ventilator patients. The early extubation strategy involved spontaneous breathing trials daily coupled with a sedation holiday on all patients who qualified (by a locally developed protocol). Peptic ulcer prophylaxis consisted of the use of famotidine, pantoprazole, or sucralfate. Hand washing was aggressively enforced by "secret shoppers" (infection control department employees masquerading as others than who they really were). Also, use of maximal barrier precautions for central line placements was mandated.

In year 2 (2005) the project was augmented with the formal addition of the concept FASTHUG to the daily patient evaluation by the critical care service. FASTHUG was considered an *evaluative *intervention; this clinical information was used to augment the procedural interventions of the previous year. During year 2 FASTHUG was emphasized on patient rounds (morning and afternoon) by the critical care team, thus allowing dissemination of the FASTHUG evaluation results in the context of a patient care plan for the day. The formal emphasis on FASTHUG at the beginning of the year 2 was not part of the original performance improvement project, but was incorporated because of the lack of significant improvement in the incidence of VAP in year 1. The CDC VAP definition was used [10].

A two-tailed z-test for two proportions was used to compare the rates for the historical period (2003) with (1) the first year of the project (2004) in which new procedural interventions were added to the care of the patient, and with (2) the second year of the project (2005), where procedural interventions were augmented with an evaluative component, FASTHUG. Follow-up pairwise comparisons were conducted using a Wilcoxon test and controlling for Type I errors across comparisons at the p = .05 level using the least significance difference (LSD) procedure. Also, interrupted time series analysis with ARIMA (auto-regressive integrated moving average) modeling [11] was then used to test for the impact of the two interventions on the monthly rates of VAP. Additionally, age, sex, race, SICU days, hospital length of stay (LOS), a severity of illness (SOI) index, and medical diagnostic categories (MDC: respiratory, circulatory, digestive, kidney/urology, and nervous systems) were compared using t test for continuous variables and Chi square test for categorical variables. SOI was determined using the risk adjustment methodology for the clinical database of the University Health System consortium [12]. P-values less than 0.05 were considered significant.

## Results

**Figure 1:**
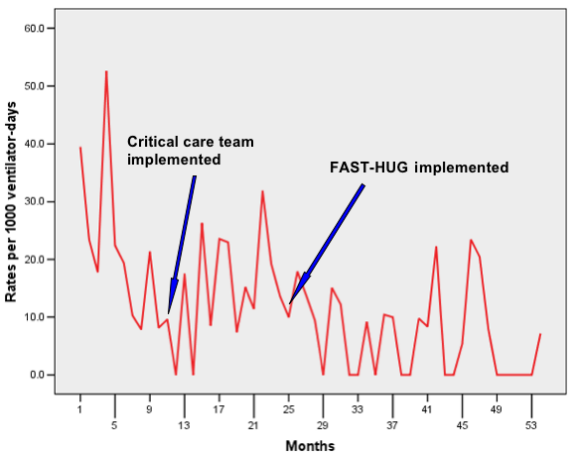
Ventilator-associated pneumonia rates over 54 months. The first twelve months are the historical period. The intensivist-led critical care team concept was implemented 60 days before year 1 of the project (month 11 of the historical period), and FAST-HUG was initiated at the beginning of year 2 of the project (month 25). A time series analysis demonstrated a significant difference in VAP rates between months 1–24 and 25–54, p = .0004. P ≤ .05 was considered statistically significant.

The SICU VAP rate for the historical control period, January–December 2003, was 19.3 VAPs/1000 ventilator-days (24 VAPs/1247 ventilator-days). The SICU VAP rate did not significantly decline in year 1 of the project (2004, pre-FASTHUG, procedural interventions only), 16.6 VAPs/1000 ventilator-days (26 VAPs/1560 ventilator-days), p = 0.62. However, with the implementation of the FASTHUG concept under the guidance of an intensivist-led critical care team, the SICU rate declined to 7.3 VAPs/1000 ventilator-days (11 VAPs/1505 ventilator-days) in year 2 (2005, post-FASTHUG), p < .01. Tables 1 and 2 demonstrate that the median pneumonia rate was significantly lower during the second year after implementing the FASTHUG concept compared to both the historical control year (z = 2.2, p = .028) and to the procedural intervention year (z = 2.04, p = .028). Daily FASTHUG evaluations then became a mainstay of critical care practice. By 30 June 2007 the VAP rate had decreased to 1.3/1000 ventilator-days (1/755 ventilator-days). In fact, there were no VAPs from January–May 2007 (Figure 1). The first time series analysis comparing the monthly rates from January–December 2003 (historical period) to those for January–December 2004 (pre-FASTHUG, i.e., addition of above-mentioned procedural interventions only) revealed no significant differences (p = 0.5909). Since the procedural interventions of 2004 produced no statistical difference in VAP rates as compared to the historical control period, the data from the historical period (2003) and the first year of the project (2004) were pooled for comparison with the 2005–2007, an extended post-FASTHUG time frame. This second time series analysis, comparing the rates from January 2003–December 2004 (months 1–24, or pre-FASTHUG) to those for January 2005–June 2007 (months 25–54, or post-FASTHUG), demonstrated a significant drop in SICU VAP rates, p = 0.0004 (Figure 1). There was no difference in age, sex, race, SICU days, LOS between, or MDC between the pre-FASTHUG time frame and the post FASTHUG time frame, although LOS trended towards significance in the post FASTHUG time frame, p = .07 (Table 3). Additionally, there was a significantly higher patient SOI index in the post-FASTHUG time frame when compared to pre-FASTHUG time frame, p = .001 (Table 3).

**Table 1:** Wilcoxon Signed Ranks Test for VAP

**Ranks**				
		**N**	**Mean Rank**	**Sum of Ranks**

**SICU Year 2 – SICU Year 1**	Negative Ranks	5 (a)	8.00	40.00
	Positive Ranks	7 (b)	5.54	38.00
	Ties	0 (c)		
	Total	12		
**SICU Year 3 – SICU Year 1**	Negative Ranks	9 (d)	7.44	67.00
	Positive Ranks	3 (e)	3.67	11.00
	Ties	0 (f)		
	Total	12		
**SICU Year 3 – SICU Year 2**	Negative Ranks	9 (g)	7.22	65.00
	Positive Ranks	3 (h)	4.33	13.00
	Ties	0 (i)		
	Total	12		

a. SICU Year 2 < SICU Year 1

b. SICU Year 2 > SICU Year 1

c. SICU Year 2 = SICU Year 1

d. SICU Year 3 < SICU Year 1

e. SICU Year 3 > SICU Year 1

f. SICU Year 3 = SICU Year 1

g. SICU Year 3 < SICU Year 2

h. SICU Year 3 > SICU Year 2

i. SICU Year 3 = SICU Year 2

VAP = ventilator-associated pneumonia

**Table 2:** Comparison of Median VAP Rates (b)

	**SICU Year 2 – SICU Year 1**	**SICU Year 3 – SICU Year 1**	**SICU Year 3 – SICU Year 2**
**Z**	-.078 (a)	-2.197 (a)	-2.040 (a)
**Asymp. Sig. (2-tailed)**	.937	.028	.041

a. Based on positive ranks.

b. Wilcoxon Signed Ranks Test

c. VAP = ventilator-associated pneumonia

**Table 3:** Descriptive characteristics of the pre- and post-FASTHUG time frames

	2003–2004 n = 1315 patients	2005–2007 n = 1653 patients	p-value
Age (mean ± sd)	58.5 ± 17.9	59.3 ± 17.2	0.23
Sex (%)			0.57
Male	40.2	39.2	
Female	59.8	60.8	
Race (%)			0.86
Whites	78.9	79.1	
Other	21.1	20.9	
LOS (mean ± sd)	13.6 ± 16.1	12.3 ± 13.2	0.07
SICU Days (mean)	7.5	7.7	0.65
MDC			0.91
Respiratory	7.88	7.14	
Circulatory	33.16	32.39	
Digestive	12.78	14.10	
Kidney/Urology	12.85	8.35	
Nervous	5.86	7.20	
SOI (%)			0.001
Minor	10.1	8.5	
Moderate	30.3	26.3	
Major	31.5	32.0	
Extreme	28.1	33.2	

LOS = hospital length of stay; SICU = surgical intensive care; MDC = major diagnostic categories; and SOI = severity of illness. P-value < 0.05 is considered statistically significant.

## Discussion

IHI 100,000 Lives Campaign has designated the prevention of VAP as one of six interventions that would significantly contribute to improved patient care and avoidable hospital deaths [6]. IHI has recommended the use of the ventilator care bundle, a group of best practices that can reduce the incidence of VAP in mechanically ventilated patients. This bundle includes deep vein thrombosis prophylaxis, stress ulcer prophylaxis, elevation of the patient's head of bed to 30–45 degrees, and a daily sedation holiday [7]. The evaluation of the sedation holiday included a spontaneous breathing trial when the patient's hemodynamic parameters were appropriate. To this bundle we added assessments of feeding, analgesia, and glucose control [9]. Additionally, this effort reinforced the Joint Commission's goal of reducing health-care associated infections.

The implementation of the FASTHUG concept by the critical care team was associated with a decreased VAP rate. However, the team's effect did not seem to be of significance in regard to VAP until a concerted effort was made to address FASTHUG on daily rounds. It can be inferred that the procedural interventions applied to the patients in year 1 were of minimal impact until the implementation of FASTHUG in year 2. The first year of the project involved changes in procedures at the bedside, whereas in the second year the changes instituted were in the evaluation of the patient. This led to examination of the patient care plan daily (sometimes even more often), allowing augmentation of the aforementioned procedural changes.

The fact that there was no difference in age, sex, race, and MDC between the pre-FASTHUG and post-FASTHUG time frames indicates that the populations were similar in their composition. However, the post-FASTHUG patients were much more ill, as indicated by their increased SOI index, p = .001. The assertion that implementation of the FASTHUG concept substantially impacted our SICU population is supported by the following: (1) the increased SOI index among the patients in the post FASTHUG time frame was coupled with a trend toward significance in the decreased patient hospital LOS, and (2) the post-FASTHUG population did not have increased lengths of stay in the SICU in the face of a higher SOI index.

Several studies support our approach. Resar et al have shown that use of a "bundle" of ventilator care processes (peptic ulcer disease prophylaxis, deep vein thrombosis prophylaxis, elevation of the head of the bed, and a sedation holiday) decreased VAP incidence by 44.5% in their intensive care unit population [13]. These authors stated that "the goal-oriented nature of the bundle appears to demand development of the teamwork necessary to improve reliability" of this approach [13]. Also, Crunden et al and Berriel-Cass et al provided evidence that VAP can be reduced by the use of "bundles" [14,15]. The former used a mandatory data collection tool for reinforcement, and the latter used the physical presence of providers to reinforce bundle compliance.

Our results may have been influenced the following factors. The use of the FASTHUG pneumonic may have created a heightened clinical acuity across disciplines regarding patient care, thereby causing more attention to be directed at the detailed care of patients. It may also be argued that better care was delivered because the parameters of FASTHUG were continually reinforced, thus facilitating a 360-degree assessment of the patient by multiple care givers. Furthermore, this study was not randomized, thus hampering our ability to make definitive conclusions. However, randomization of patients to receive or not to receive good care that has been recognized by IHI and JCAHO may be unethical. Another difficulty is that our study was monocentric and was conducted in a small university medical center. These conditions could create bias regarding the types, complexity, and number of cases that were treated. Clearly any conclusions from this small observational and retrospective study should be done cautiously. Larger studies that are multi-centered and prospective need to be performed to better assess our results. Finally, the improvement reported in our SICU VAP rates may have occurred simply because there was a critical care team present to provide care. In attempting to analyze the data it may be difficult to separate FASTHUG implementation from good critical care practice.

The FASTHUG application may not apply to all patients at all times, but its daily reappraisal at the SICU patient's bedside at our institution allowed implementation of a strategy that reinforced teamwork and improved patient care. All members of the patient care team understood what the pneumonic FASTHUG represented, and its importance to patient quality of care and safety. While this study may only have been observational in its methodology, the implementation of daily FASTHUG evaluations at the bedside produced a significant effect on the VAP rate in our SICU patients.

## Competing interests

The author(s) declare that they have no competing interests.

## Authors' contributions

TJP, SJH, and JMD conceived of the idea. TJP, SJH, and LRO collected the data. TJP, MJB, and JJF cared for the patients. TJP, SJH, JMD, SAK, MJB, and JJF edited the manuscript. SAK, TJP, and DO were responsible for the statistical analysis. All authors have read and approved the final manuscript.
